# The Expanding Mycovirome of *Aspergilli*

**DOI:** 10.3390/jof10080585

**Published:** 2024-08-17

**Authors:** Josephine L. Battersby, David A. Stevens, Robert H. A. Coutts, Vladimír Havlíček, Joe L. Hsu, Gabriele Sass, Ioly Kotta-Loizou

**Affiliations:** 1 Department of Life Sciences, Imperial College London, London SW7 2AZ, UK; 2 Department of Clinical, Pharmaceutical and Biological Science, University of Hertfordshire, Hatfield AL10 9AB, UK; j.l.battersby@herts.ac.uk (J.L.B.); r.coutts@herts.ac.uk (R.H.A.C.); 3 California Institute for Medical Research, San Jose, CA 95128, USA; stevens@stanford.edu (D.A.S.); gcmsass@googlemail.com (G.S.); 4 Division of Infectious Diseases and Geographic Medicine, Stanford University School of Medicine, Stanford, CA 94305, USA; 5 Institute of Microbiology of the Czech Academy of Sciences, Videnska 1083, 142 00 Prague, Czech Republic; vlhavlic@biomed.cas.cz; 6 Department of Analytical Chemistry, Palacky University, 17. Listopadu 2, 779 00 Olomouc, Czech Republic; 7 Department of Medicine, Division of Pulmonary, Allergy and Critical Care Medicine, Stanford University School of Medicine, Stanford, CA 94305, USA; joehsu@stanford.edu

**Keywords:** mycovirus, *Aspergillus*, *Polymycovirus*, RNA sequencing, hypovirulence, oxidative stress, mycovirus-host interactions, aspergillosis, *Pseudomonas aeruginosa*, mycotoxins, RNA silencing, antifungal treatment, phage therapy

## Abstract

Mycoviruses are viruses that infect fungi and are widespread across all major fungal taxa, exhibiting great biological diversity. Since their discovery in the 1960s, researchers have observed a myriad of fungal phenotypes altered due to mycoviral infection. In this review, we examine the nuanced world of mycoviruses in the context of the medically and agriculturally important fungal genus, *Aspergillus*. The advent of RNA sequencing has revealed a previous underestimate of viral prevalence in fungi, in particular linear single-stranded RNA viruses, and here we outline the diverse viral families known to date that contain mycoviruses infecting *Aspergillus*. Furthermore, we describe these novel mycoviruses, highlighting those with peculiar genome structures, such as a split RNA dependent RNA polymerase gene. Next, we delineate notable mycovirus-mediated phenotypes in *Aspergillus*, in particular reporting on observations of mycoviruses that affect their fungal host’s virulence and explore how this may relate to virus-mediated decreased stress tolerance. Furthermore, mycovirus effects on microbial competition and antifungal resistance are discussed. The factors that influence the manifestation of these phenotypes, such as temperature, fungal life stage, and infection with multiple viruses, among others, are also evaluated. In addition, we attempt to elucidate the molecular mechanisms that underpin these phenotypes, examining how mycoviruses can be targets, triggers, and even suppressors of RNA silencing and how this can affect fungal gene expression and phenotypes. Finally, we highlight the potential therapeutic applications of mycoviruses and how, in an approach analogous to bacteriophage therapy, their ability to produce hypovirulence in *Aspergillus* might be used to attenuate invasive aspergillosis infections in humans.

## 1. Introduction

**Figure 1:**
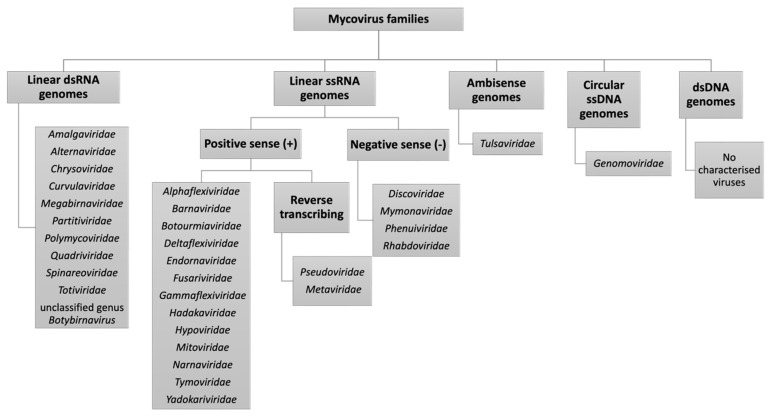
An overview of the 31 viral families (and one unclassified genus) known to infect fungi. Genomes of the families can be linear double stranded RNA, positive-sense (+) single stranded (ss) RNA (including reverse transcribing viruses), negative-sense (−) ssRNA, ambisense, or circular ssDNA. No mycoviruses with dsDNA genomes have been isolated.

Viruses are infectious agents to which all life forms are susceptible, and fungi are no exception. Mycoviruses, first reported 72 years ago in the edible mushroom *Agaricus bisporus* [1], are viruses that infect fungi and are widespread across all major fungal taxa, exhibiting vast biological diversity [2,3]. At present, the International Committee on Taxonomy of Viruses (ICTV) recognises 31 mycovirus families and one unclassified genus, in addition to numerous unassigned viruses awaiting classification [4,5] (https://talk.ictvonline.org/taxonomy/, accessed on 5 June 2024) (Figure 1). The majority of identified mycoviruses have (i) linear double-stranded (ds) RNA genomes, belonging to the families *Amalga*-, *Alterna*-, *Chryso*-, *Curvula*-, *Megabirna*-, *Partiti*-, *Polymyco*-, *Quadri*-, *Spinareo*-, *Totiviridae*, and the unclassified genus *Botybirnavirus;* or (ii) linear positive-sense, single-stranded RNA (+ssRNA) genomes, belonging to the families *Alphaflexi*-, *Barna*-, *Botourmia*-, *Deltaflexi*-, *Endorna*-, *Fusari*-, *Gammaflexi*-, *Hadaka*-, *Hypo*-, *Mito*-, *Narna*-, *Tymo*-, *Yadokari*-, and the reverse transcribing (RT) *Pseudo*- and *Metaviridae.* Less common are mycoviruses with linear, single stranded (ss) negative-sense ssRNA (–ssRNA) genomes, comprising the families *Disco*-, *Mymona*-, *Phenui*- and *Rhabdoviridae.*

The +ssRNA viruses act as mRNA and can be directly translated by host machinery, whereas –ssRNA viruses are the complement of mRNA and must be converted to +ssRNA by RNA-dependent RNA polymerase (RdRP) prior to translation. Genomes of the RT viruses, however, are positive-sense but replicated by a reverse transcriptase to generate complementary DNA, which is in turn inserted into the host genome *via* a viral encoded integrase enzyme [6,7]. In addition, viruses with ambisense genomes, containing both positive-sense and negative-sense RNAs, have been isolated from fungi, and belong to the family *Tulsaviridae* [8]. Lastly, a small number of mycoviruses with circular ssDNA genomes, from the family *Genomoviridae*, have been reported [9,10]. To date, no mycoviruses with dsDNA genomes have been fully molecularly characterised.

The advent of high-throughput, next-generation sequencing (NGS), in particular deep RNA sequencing (RNA-seq), has revolutionised the field of mycovirology, facilitating the rapid detection of viruses from diverse fungal hosts and elucidating their evolutionary origins [11,12,13,14]. Remarkably diverse genome structures, replication strategies and lifecycles have been observed across the mycoviral lineages [5]. Genomes of dsRNA mycoviruses, except for *Toti*- and *Amalgaviridae*, are multi-segmented, range in length from 3–29 kbp, and encode up to 12 genes [3]. Excluding members of the genus *Botybirnavirus* and family *Polymycoviridae*, dsRNA mycoviruses usually encode a capsid protein (CP), encapsidating their genomes into isometric capsids, with each RNA segment typically packaged into its own particle [15]. Conversely, ssRNA mycovirus genomes, which range from 2–17.6 kbp, are usually unsegmented [16], excluding the newly classified *Hadakaviridae* family [17]. Furthermore, many +ssRNA mycoviruses, such as those of the *Narnaviridae* family, are capsidless and do not form true virus particles [18]. A ubiquitous feature of all RNA mycoviruses, excluding RT viruses, is the presence of RdRP for genome replication [19]. Lastly, the ssDNA mycoviruses have smaller, circular genomes (1.3–2.4 kbp) packaged in isometric virions [9] and are circular rep-encoding single-stranded (CRESS) viruses, meaning they encode a replicase (rep) and a CP [20]. Construction of mycoviral taxonomy by the ICTV is predominantly based on molecular phylogeny, but also incorporates genome characteristics and biological properties [21,22] and requires constant updating. Importantly, while research has focused on RdRPs and CPs, functions of other mycoviral proteins remain ambiguous and should be researched further to better understand mycovirus biology.

From a public health stance, especially in a post-SARS-CoV-2 pandemic landscape, associations with viruses tend to be negative. Refreshingly, mycoviruses have attracted attention for the opposite reason. Mycovirus infections are persistent and typically cryptic [23]–however, some have been found to cause decreased virulence (hypovirulence) in their fungal host [24]. This phenomenon was first observed during the 1970’s in the chestnut blight fungus *Cryphonectria parasitica*, a phytopathogen that causes significant agricultural losses to American and European populations of chestnut trees. The presence of a mycovirus, Cryphonectria hypovirus 1 (CHV1), in *C. parasitica* significantly reduced the fungal virulence, exhibiting a protective effect on the trees. Consequently, CHV1-infected *C. parasitica* strains were successfully employed as biological control agents to manage outbreaks of the fungus across Europe [25,26]. Following this discovery, mycoviruses have been of interest for their potential use in biocontrol and, while this has focused on phytopathogenic fungi for agricultural and ecological applications [27], mycoviruses that elicit hypovirulence in human pathogenic fungi are now being explored, alluding to potential therapeutic applications [28].

In addition to hypovirulence, other reported mycovirus-mediated phenotypes include increased virulence (hypervirulence) [29,30,31] and effects on sporulation [32,33], growth, colony morphology [34], pigmentation [35], secondary metabolite and toxin production [36,37,38,39,40] drug resistance [41,42], control of endophytic traits [43,44] and biotic/abiotic stress tolerance [45,46,47]. Manifestation of these mycovirus-phenotypes may be modulated by abiotic and biotic factors [33,48]. Remarkably, in addition to phenotypic changes in the host fungus, mycoviruses can affect interactions between the virus-infected fungus and an organism hosting that fungus, i.e., an insect, plant, or animal [30].

The majority of mycoviruses lack an extracellular stage in their lifecycle and are transmitted intracellularly, either (i) horizontally, through hyphal anastomosis, or (ii) vertically, *via* sexual and asexual spore dissemination. Hyphal anastomosis, which involves hyphal fusion and exchange of cytoplasmic materials (including mycovirus particles), can only occur if strains are vegetatively compatible [16,49]. The fungal vegetative incompatibility genes (*vic*) regulate compatibility during hyphal anastomosis by initiating programmed cell death upon contact between incompatible fungi [50,51]. Vegetative incompatibility presents a hurdle in the application of mycoviruses as biocontrol agents and must be overcome to harness their potential [51]. For example, while the use of CHV1-infected *C. parasitica* was successful in Europe, this approach failed in the United States due to vegetative incompatibility between strains [52]. Interestingly, plants have been reported to play a role in horizontal virus transmission within vegetatively incompatible strains of *Sclerotinia sclerotiorum*, enhancing mycovirus transmission efficiency *via* a mechanism that involves proline accumulation [53].

*Aspergillus* is a genus of fungi (phylum: Ascomycota, class: Eurotiomycetes, order: Eurotiales) comprising over 250 species of filamentous fungi [54]. Species of the genus, which thrive in oxygen-rich environments, have a global distribution and are among the most extensively researched fungi because of their medical, commercial, and ecological relevance. For example, the human pathogen *Aspergillus fumigatus* is the primary cause of aspergillosis, a group of potentially life-threatening respiratory diseases [55], while the plant and human pathogen *A. flavus*, as well as also being a cause of aspergillosis, is responsible for significant agricultural losses *via* crop infection and production of the potent carcinogenic metabolite, aflatoxin [56]. Furthermore, species *A. oryzae* and *A. sojae* are employed commercially in the production of fermented foods (e.g., soy sauce), while *A. terreus* and *A. niger* are used industrially in the production of organic acids and enzymes, respectively [57,58].

Mycoviruses in *Aspergilli* have been documented, with diverse mycoviral lineages observed across the genus, as well as a range of peculiar virus-mediated host phenotypes. As research in the field accelerates, this review aims to present an update on mycoviruses in *Aspergilli*, examining recently characterised novel mycoviruses and any associated virus-induced phenotypes, unearthing the molecular mechanisms that underpin these host-virus interactions, and discussing potential mycovirus-based therapeutic applications.

## 2. Characterisation of Novel Mycoviruses in *Aspergilli*

Since the first observation of mycoviruses in *Aspergilli* in 1970 [59], screening of *Aspergillus* isolates for mycoviruses has been extensive, with numerous viruses characterised across a range of species. The incidence of mycoviruses in *Aspergilli* was reviewed by Kotta-Loizou and Coutts in 2017 [60], who provided a thorough summary of population studies. A prevalence of dsRNA mycoviruses in species of the *Aspergillus* sections Nigri, Circumdati, Flavi, Clavati and Fumigati that ranged from 7–50% was reported, as observed in strains isolated from worldwide locations. At the time of the 2017 review, characterised *Aspergilli* mycoviruses belonged to the families *Partiti*-, *Toti*-, *Chryso*-, *Polymyco*- and *Alternaviridae*. Since then, however, knowledge of the *Aspergilli* virome has broadened, with the recent discovery of novel viruses belonging to a wider range of viral families and isolated from more diverse fungal hosts. Here, we report the viral families known to date that contain mycoviruses infecting *Aspergillus* (Table 1) and describe in depth, to the best of our knowledge, the viruses identified and characterised since 2017. Table S1 (Supplementary Material) outlines these viruses, along with providing a summary of the known viral properties. Due to space limitations and the increasingly extensive isolation of virus-like particles *via* metagenomics, our review may not be exhaustive, and we have prioritised describing viruses with peculiar or notable genome organisations in the text.

### 2.1. The First Observations of ssRNA Viruses in Aspergilli

#### 2.1.1. *Narnaviridae*

Previously, detection of mycoviruses relied on the extraction of dsRNA viral genomes or dsRNA replicative intermediates, which can be visualised by agarose gel electrophoresis (AGE) and purified to construct cDNA libraries for sequencing [61]. Unfortunately, this method led to an under-representation of ssRNA mycoviruses due to its higher efficiency for the isolation of dsRNA viral genomes as compared to replicative intermediates [62]. Until recently only dsRNA mycoviruses had been observed in *Aspergilli*, but with the advent of deep RNA-seq technologies, this is no longer the case.

The *Narnaviridae* are a family of simple viruses, which consist of unencapsidated +ssRNA genomes (2.3–2.9 kbp), comprising a single open reading frame (ORF) that encodes the RdRP [18]. The family has a single genus, *Narnavirus*, and fungi serve as natural hosts. RNA-seq was employed by Zoll et al. [62], which involved mapping sequencing reads from *A. fumigatus* strains against a reference genome and collecting unmapped reads for *de novo* assembly of contigs. The Basic Local Alignment Search Tool x (BLASTx) was then used to analyse contigs and detect mycoviruses. The authors detected two contigs, both of which represented single ORFs encoding narnavirus RdRPs, designating them Aspergillus fumigatus narnavirus 1 (AfuNV1) and Aspergillus fumigatus narnavirus 2 (AfuNV2), and providing the first report of ssRNA mycoviruses in *Aspergilli*.

RNA-seq technologies are restricted by their inability to isolate viral genomes that have no homology to known mycoviral sequences [63]. Fragmented and Primer-Ligated Double-stranded RNA Sequencing (FLDS) is a technology that facilitates the detection of mycoviral genomes in a homology-independent manner. Application of this technology by Chiba et al. [63] revealed that in some cases the narnavirus RdRP gene is divided among viral segments. This was true for the recently isolated AfuNV2, contrasting with the findings by Zoll et al. [62] and the previously accepted view that RdRPs are encoded by a single gene. Narnaviruses with non-divided RdRPs contain five conserved motifs (A, B, C, D and F). Chiba et al. [63] observed that the RdRP of AfuNV2 is encoded by two ORFs located on separate viral segments, one of which contains motifs F, A and B and the other motifs C and D. FLDS has also been used to detect additional narnaviruses with divided RdRPs, including Aspergillus lentulus narnavirus 1 (AleNV1), from *A. lentulus*, an opportunistic human pathogen commonly resistant to key clinical antifungals [63], and Aspergillus tennesseensis narnavirus 1 (AtenNV1), from deep-sea strain *Aspergillus tennesseensis* [64]. The divided RdRPs of AleNV1 and AfuNV2 are classified as ‘type I’ because they comprise the same division site, between domains B and C. Meanwhile, a ‘type II’ divided RdRP, was observed in AtenNV1, with the protein split at a site between domains A and B. In both types of division, an active protein is thought to form *via* binding of the two segments with hydrophobic and van der Waals forces [64].

Recently, the name ‘*Splipalmiviruses*’ has been coined for narna-like-viruses that encode an RdRP with a split palm domain, *spli*(t)*palmivirus*, meaning one segment encodes motifs F, A, and B and the other motifs C and D [65]. An RNA-seq study in *A. flavus* reported a novel trisegmented narnavirus, designated Aspergillus flavus narnavirus 1 (AfNV1) [66], and a BLASTp search of each segment showed high degrees of amino acid (aa) similarity with other narnavirus RdRPs. This may suggest that AfNV1 RdRP is divided over three segments. Interestingly, each segment shared homology with another trisegmented splipalmivirus, Beavueria bassiana splipalmivirus 1 (BbPV1), suggesting AfNV1 may also be splipalmiavirus [66,67].

#### 2.1.2. *Mitoviridae*

Previously, *Narnaviridae* comprised two genera, *Narnavirus* and *Mitovirus*. These genera were delineated by their replication site within the fungal host, with replication of the former occurring in the cytoplasm and the latter in the mitochondria [18]. As well as employing different cellular machinery for their replication, mitoviruses uniquely translate their genome using the fungal mitochondrial genetic code, whereby internal UGA codons encode tryptophan instead of a stop codon [68]. The differences in their replication styles, in combination with the generation of more extensive RNA-seq-based phylogenetic trees, led to the re-classification of *Mitoviridae* into its own family containing the genus *Mitovirus* [68,69]. Aspergillus fumigatus mitovirus 1 (AfuMV1) is a novel mycovirus isolated from *A. fumigatus via* RNA-seq [62]; the virus was originally classified as a member of the *Narnaviridae* but now falls within the *Mitoviridae*.

#### 2.1.3. *Botourmiaviridae*

*Botourmiaviridae* is a family of +ssRNA viruses, from which the genera *Botoulivirus*, *Magoulivirus*, *Scleroulivirus* and *Ourmiavirus* are known to infect fungi. The genomes of these genera are unencapsidated, monosegmented (2–5 kbp), and solely encode an RdRP [70]. Using RNA-seq several members of the *Botourmiaviridae*, comprising monosegmented RdRP encoding genomes have also been isolated from *Aspergilli* (Table S1) [28,63,66]. Notably, one of these viruses, Aspergillus creber ourmiavirus 1 (AcreOV1), from the genus *Ourmiavirus*, was found to have two RNA segments, the second of which did not encode a known protein [64].

### 2.2. Expansion of the dsRNA Virome

The presence of dsRNA mycoviruses in *Aspergilli* is well-established. To our knowledge, no novel viruses from the family *Alternaviridae* have been reported in the genus since the last review [60]. Briefly, members of the *Alternaviridae* infect exclusively ascomycetes and possess 8.4–10.7 kbp genomes with three or four dsRNA segments, which harbour poly A tails at the 3′-terminus [71,72,73,74,75,76,77]. However, numerous novel mycoviruses from the dsRNA families *Partiti*-, *Chryso*-, *Toti*- and *Polymycoviridae* have been recently identified in *Aspergillus* and are discussed here in detail.

#### 2.2.1. *Partitiviridae*

The *Partitiviridae* family comprises viruses with small (3–4.8 kbp), non-enveloped and bisegmented dsRNA genomes, in which each segment is packaged separately in an isometric virion [78]. The family constitutes five genera, of which predominantly *Alpha*-, *Beta*-, *and Gammapartitivirus* have been reported in fungi. RNA-seq has facilitated the isolation of numerous new members of the *Partitiviridae.* As such, Zoll et al. [62] identified the novel Aspergillus fumigatus partitivirus 2 (AfuPV2) from *A. fumigatus*, detecting both segments, encoding the putative RdRP and CP, of the characteristic bisegmented genome. The 5′ termini end of the segments showed high similarity to the conserved 5′ untranslated regions (UTR) of two other partitiviruses, Alternaria alternata partitivirus 1 (AtPV1) and Botryosphaeria dothidea partitivirus 1 (BdPV1). Phylogenetic analysis placed AfuPV2 in a clade with AtPV1 and BdPV1, forming an intermediate between *Gamma*- and *Deltapartitivirus*. The RdRP and CP of AfuPV2 have an 84% and 71% aa similarity to those respective proteins of another recently identified *Aspergillus* partitivirus, namely Aspergillus lentulus partitivirus 1 (AlePV1), isolated from *A. lentulus* by FLDS [63]

While partitiviruses typically comprise two dsRNA segments, the larger of which encodes the RdRP and the smaller the CP, an additional third dsRNA has occasionally been reported [79]. Aspergillus fumigatus partitivirus 1 (AfuPV1) is a previously described partitivirus from *A. fumigatus*, which was thought to comprise just two segments [80]. However, Filippou et al. [81] isolated a trisegmented AfuPV1 from a Portuguese *A. fumigatus* strain. With dsRNA1 and dsRNA2 respectively encoding a putative RdRP and CP, the smallest fragment (dsRNA3) encodes a protein of unknown function. BLASTp indicated that the hypothetical protein sequence had the highest homology (35.6%) to a corresponding segment with unknown function from another trisegmented partitivirus, Trichoderma harzianum partitivirus 3. The 5′ UTRs of all three segments possessed conserved nucleotide sequences. In partitiviruses, conserved nucleotides at the 5′ terminus may facilitate RdRP recognition for RNA packaging and virus replication [78,81]. Moreover, considering that AfuPV1 can replicate in the absence of dsRNA3, it is possible that the protein is defective but continues to be replicated as it contains the conserved 5′ terminus sequence. That said, until reverse genetics studies are conducted to investigate this hypothesis, the role of the protein remains ambiguous.

A novel trisegmented partitivirus was also identified by Jiang et al. [82], isolated from *A. flavus* and designated Aspergillus flavus partitivirus 1 (AfPV1). The AfPV1 dsRNA3 shares no homology with any sequences in the GenBank database; its function also remains unknown. Phylogenetic analysis of AfPV1 placed the virus in an unclassified cluster with several other partitiviruses, and the authors proposed the new genus, ‘Zetapartitivirus’. Aspergillus niger partitivirus 1 (AnPV1), another new partitivirus isolated from *A. niger* by NGS, shows 78% RdRP aa similarity to AfPV1 and may also fall within the ‘Zetapartitivirus’ [28]. Interestingly, a partitivirus, recently detected in *A. flavus via* FLDS, has high aa sequence homology to AfPV1 but contains a total of eight dsRNA segments [83]; dsRNA1, 2, 3, 4 and 5 shared aa sequence homologies with AfPV1, while dsRNA6, 7 and 8 displayed no sequence similarity with AfPV1 [83].

Some mycoviruses have been found to possess additional subviral agents, known as satellite viruses or satellite nucleic acids [84,85,86,87]. These satellite viruses are dependent on the co-infecting virus, known as the ‘helper virus’, for their infection cycles. Satellite viruses encode their own CP, whereas satellite nucleic acids rely on structural proteins encoded by the helper virus for encapsidation. Following their earlier study [82], Jiang et al. [88] isolated a helper AfPV1 virus associated with a small dsRNA (734 bp) thought to be a satellite RNA. The segment contained no ORF, thus not encoding a protein, and was thought to employ the AfPV1 CP for its encapsidation, deeming it a satellite RNA, rather than satellite virus. Importantly, helper AfPV1 virus could replicate without the satellite dsRNA, but not *vice versa*, unlike AfPV1 dsRNA3 as described by Jiang et al. [82]. The satellite and helper virus harboured conserved nucleotides at their 5′ termini, with a similar sequence found in BdPV1. Furthermore, the secondary structure of over half of the satellite’s nucleotides were involved in the construction of stem loops. Both the conserved sequences and the stem loop structures may relate to a RdRP recognition and replication functionality [89,90].

Isolated from the species *A. nidulans*, a model organism and versatile enzyme-producing cell factory [91], Aspergillus nidulans partitvirus 1 (AnPV1) is a novel partitivirus that also falls phylogenetically outside of the established *Partitiviridae* genera [92]. Phylogenetic analysis based on both RdRP and CP sequences placed the bisegmented virus in an unclassified clade between the proposed ‘Zetapartitivirus’ and *Deltapartitivirus*, suggesting it belongs to an additional unclassified genus. Another novel partitivirus, Aspergillus creber partitivirus 1 (AcrePV1), identified from deep-sea *A. creber* strains *via* FLDS [64], had the highest degree of RdRP aa similarity (68%) to AnPV1, and may fall within the same cluster.

#### 2.2.2. *Chrysoviridae*

*Chrysoviridae* are a family of small (8.9–16 kbp), non-enveloped viruses, comprising dsRNA genomes of three to seven segments, each encapsidated in individual isometric particles [93]. The family consists of two genera, *Alpha*- and *Betachrysovirus*, both of which infect fungi. Lutz et al. [94] identified a novel trisegmented chrysovirus from *A. cibarius*, designated Aspergillus cibarius chrysovirus 1 (AcCV1). The RdRP, encoded by the largest segment, has a 99.1% similarity to the RdRP of the Hulunbuir Chrys tick virus 1 (HCTV1). As hypothesised by the authors, this high degree of similarity could suggest that HCTV1 is not a tick virus but instead a mycovirus that was present in the insect due to a fungal infection. Protein sequencing indicated that the second and third segments encode individual proteins which together may form the capsid and a discrepancy between their calculated protein sizes and their AGE band size suggests that they may undergo post-translational modifications [94]. Finally, the authors proposed the new genus ‘Gammachrysovirus’ because the virus forms a novel phylogenetic cluster with HCTV1 separate from *Alpha*- and *Betachrysovirus.*

From the human pathogen *A. thermomutatus*, a betachrysovirus comprising four segments, each encoding a single ORF, was identified and named Aspergillus thermomutatus chrysovirus 1 (AthCV1) [32]. With a putative RdRP and CP encoded by the first and second largest segments, respectively, the functions of the proteins encoded by the two smallest segments are unknown, though shared homology with other hypothetical chrysovirus proteins. Furthermore, a novel trisegmented alphachrysovirus, Aspergillus terreus chrysovirus 1 (AtCV1), has been reported in *A. terreus* [95], a species employed industrially to produce secondary metabolites, and a human pathogen, one regarded as often resistant to many available antifungals [96,97]. Similarly to AthCV1, the two largest segments of AtCV1 encode an RdRP and CP, while the function of the proteins encoded on the smallest two segments are unknown. In many chrysoviruses, the 5′ UTR of each segment harbours a highly conserved A-rich 40–75 nucleotide sequence, as well as a second conserved region downstream that comprises repeats of the translational enhancer sequence ‘CAA’ [93,98]. Despite AtCV1, AthCV1 and AcCV1 all possessing highly conserved chrysovirus 5′ UTRs, the ‘CAA’ repeats were only observed in segments of AtCV1, suggesting this feature may not necessarily be characteristic of *Chrysoviridae* [94].

Also possessing highly conserved 5′ and 3′ UTR terminal sequences, whilst lacking ‘CAA’ repeats, is the recently isolated Aspergillus fumigatus chrysovirus 41362 (AfuCV41362) [99]. Conservation of UTR nucleotide sequences is a typical characteristic of multisegmented RNA viruses and indicates that each segment replicates itself separately [99,100]. The AfuCV41362 genome harbours four dsRNA segments, each comprising a single ORF (ORFs 1–4). The aa sequence of each segment shares homology with corresponding proteins from other chrysoviruses, with ORF2, ORF3 and ORF4 showing the highest degrees of similarity (45–68%) to the respective proteins of Penicillium janczewskii chrysovirus 1, from *P. janczewskii*. Meanwhile, ORF1 has an identical sequence to a virus originally designated as Aspergillus mycovirus 1816 (AsV1816) from *A. nidulans* [73], which is now fully cloned and sequenced. ORF1 and ORF3 respectively encode a putative RdRP and CP, while ORF2 and ORF4 encode hypothetical proteins with unknown function.

#### 2.2.3. *Totiviridae*

Viruses belonging to the family *Totiviridae* are packaged in isometric virions and comprise a monosegmented genome, 4.6–6.7 kbp, encoding both an RdRP and CP, which are usually expressed *via* ribosomal frameshifting [101]. The family contains five genera, of which *Totivirus* and *Victorivirus* infect exclusively fungi. A novel virus, phylogenetically identified as a victorivirus, was isolated from *A. niger* and designated Aspergillus niger victorivirus 1 (AnV1) [102]. The AnV1 genome harbours two ORFs, overlapping at a tetranucleotide sequence (AUGA), in which ORF1 (putative RdRP) stop codon overlaps with ORF2 (putative CP) start codon [102]. This tetranucleotide is a common motif in victoriviruses, facilitating the translation of a downstream ORF2 *via* a termination-reinitiation mechanism [102,103].

#### 2.2.4. *Polymycoviridae*

Viruses from the *Polymycoviridae* family possess non-conventionally encapsidated, multisegmented dsRNA genomes (7.5–12.5 kbp), comprising four to eight segments, and fungi and oomycetes serve as natural hosts [104]. The family accommodates one genus, *Polymycovirus.* Polymycoviruses provided the first report of dsRNA viruses that are infectious as naked dsRNA [105]. Aspergillus fumigatus polymycovirus 1 (AfuPmV1) is the prototype member of the family and was originally named Aspergillus fumigatus tetramycovirus 1 (AfuTmV1), prior to the discovery of similar viruses comprising additional segments [60,105]. Despite having been reviewed previously [60], we discuss AfuPmV1 here due to its importance in mediating *Aspergillus* phenotypes (see Section 3, ‘Mycovirus-mediated phenotypes’).

Polymycovirus RdRPs uniquely possess a conserved aa GDNQ motif, which is usually found in –ssRNA viruses and differs from the GDD motif typically found in dsRNA viruses [33]. This is the case for AfuPmV1 RdRP, encoded by the largest segment. The function of the protein encoded by the second largest segment is unknown, containing a zinc finger-like motif as well as arginine repeats linked to endoplasmic reticulum (ER) retention signalling [33]. An *S*-adenosyl methionine-dependent methyltransferase, involved in capping the 5′ends of positive sense strands and typically encoded by the same segment that encodes the RdRP, is instead encoded by the third largest segment of AfuPmV1 [105]. Lastly, a proline-alanine-serine (PAS) rich protein is encoded by the smallest segment, which putatively coats the viral genome, without encapsidating it. BLASTp revealed that all four AfuPmV1 segments share high degrees of aa similarity (63–73%) to a novel tetra-segmented polymycovirus from *A. spelaeus*, namely Aspergillus spelaeus polymycovirus 1 (AsPmV1) [106].

A polymycovirus, which displayed four segments with very high aa sequence similarity to AfuPmV1 (95–98%), but containing an additional fifth dsRNA segment, has also been isolated from *A. fumigatus* [107]. The fifth segment was the second smallest and displayed no similarity to any other sequence in GenBank; it is possible the segment may have been evolutionarily lost from AfuPmV1. The virus was designated Aspergillus fumigatus polymycovirus 1M (AfuPmV1M). Additionally, a novel dsRNA virus, harbouring four segments each encoding the characteristic corresponding polymycovirus proteins (RdRP, methyl-transferase protein, PAS-rich protein and hypothethical polymycovirus protein with unknown function) has also been isolated from *A. flavus via* NGS and designated Aspergillus flavus polymycovirus 1 (AfPMV1) [66].

**Table 1:** Viral families containing mycovirus that infect Aspergillus. Characteristics, including genome type and length, encapsidation, are provided, along with an example of a virus from the family in *Aspergillus*.

Family	Genome Type	Genome Length (kbp)	Segmentation *	Encapsidation	Example of Mycovirus in *Aspergillus*
***Narnaviridae***	Linear (+) ssRNA	2.3–2.9	Unsegmented	Unencapsidated	Aspergillus fumigatus narnavirus 1 (AfuNV1) [62]
***Mitoviridae***	Linear (+) ssRNA	2.5–2.9	Mono-segmented	Unencapsidated	Aspergillus fumigatus mitovirus 1 (AfuMV1) [62]
***Botourmiaviridae***	Linear (+) ssRNA	2.9–5	Monosegmented **	Unencapsidated **	Aspergillus fumigatus botourmiavirus 1 (AfuBOV1) [28]
***Partitiviridae***	Linear dsRNA	3–4.8	Bi- or tri-segmented	Encapsidated	Aspergillus fumigatus partitivirus 1 (AfuPV1) [80,81]
***Totiviridae***	Linear dsRNA	4.6–6.7	Mono-segmented	Encapsidated	Aspergillus niger victorivirus 1 (AnV1) [102]
***Chrysoviridae***	Linear dsRNA	8.9–16	Four segments	Encapsidated	Aspergillus fumigatus chrysovirus 41,362 (AfuCV41362) [99]
***Polymycoviridae***	Linear dsRNA	7.5–12.5	Four segments	Unconventionally encapsidated	Aspergillus fumigatus polymycovirus 1 (AfuPMV1) [60,105]
***Alternaviridae***	Linear dsRNA	8.4–10.7	Three to four segments	Encapsidated	Aspergillus foetidus alternavirus (AfAV) [108]

* Viruses from these families with additional segments to the number displayed here have been isolated. ** genus *Ourmiavirus* is multisegmented and encapsidated.

## 3. Mycovirus-Mediated Phenotypes

‘La France’ was a disease first observed in 1948 in the cultivated mushroom *A. bisporus*, characterised by slow and aberrant mycelial growth and the premature emergence or malformation of fruiting bodies [109]. It was later established that the cause of this agriculturally detrimental disease was, in fact, a mycovirus, namely Agaricus bisporus virus 1 (AbV1) [110]. Since this discovery, numerous altered phenotypes attributable to mycovirus infection have been observed across the Fungi kingdom. Some mycovirus-mediated phenotypes have even led to exciting scientific breakthroughs. For example, mycoviruses identified in the commercial yeast *Saccharomyces cerevisiae*, are known to cause the production of extracellular toxins that eliminate competing yeasts, activating the ‘killer yeast’ phenotype [111,112]. This phenotype is produced by M satellite dsRNAs (M1, M2, M28), encoding respective toxins (K1, K2, K28), which depend on a helper totivirus L-A. Killer yeasts have been exploited widely in industry for food preservation and biocontrol of spoilage organisms [113].

While metagenomics has facilitated the rapid identification of mycoviruses, phenotypic screening of the fungal host presents a bottleneck in research. This is because reliably investigating the phenotypic effect of a virus requires the generation of isogenic fungal strains that deviate solely in their possession of the virus of interest [114]. Unfortunately, RNA mycoviruses lack infection machinery to facilitate extracellular transmission, thus the isogenic fungal strains must be artificially produced, either by (i) curing the virus from an infected strain, or (ii) infecting a virus-free recipient strain with the purified virus [115].

As reviewed by Khan et al. [116], methods of mycoviral curing include: protein synthesis inhibitors (e.g., cycloheximide) [117], antiviral compounds (e.g., ribavirin) [118], hyphal tipping [119], single-spore isolation [99,107], heat stress, and several other strategies. Conversely, virus infection involves protoplast transfection or protoplast fusion [60]. When undertaking either approach, researchers must ensure that the original isolates contain clonal populations of nuclei and, if employing a curing protocol, cured strains should be re-infected with the virus to confirm that phenotypes are caused by the virus rather than effect of the compounds involved in the process [60,114]. Notably, the efficiency of mycoviral curing can vary depending on the fungal strain, viral species and the method employed [115,116]. A comparative study by Ikeda et al. [115], in which *A. fumigatus* strains were cured using the antiviral compounds ribavirin, 2′-C-methylcytidine (2CMC), 2′-C-methyladenosine (2CMA), and 7d2CMA, reported that 2CMC had the highest virus-curing efficacy. Finally, researchers should consider the possibility that an artificially re-infected strain may behave differently to naturally virus-infected strain, impacting the validity of experimental results [114].

Following the production of isogenic strains, phenotypic screening protocols, ranging from conventional to more advanced, are conducted, with pigmentation, growth, virulence, sporulation, and toxin production most examined. For example, traditionally fungal growth rates were measured by colony diameter or mycelial biomass; more recently, this phenotype has been quantified using XTT assays, which employ a tetrazolium salt (XTT), to measure fungal metabolism [120]. Virulence in vivo is typically determined using murine [99,107] or insect models [121], in which mortality rates and fungal burden are recorded following inoculation with the fungi of interest. Finally, RNA-seq has been increasingly employed to examine differential gene expression in mycovirus-infected fungi. 

**Figure 2:**
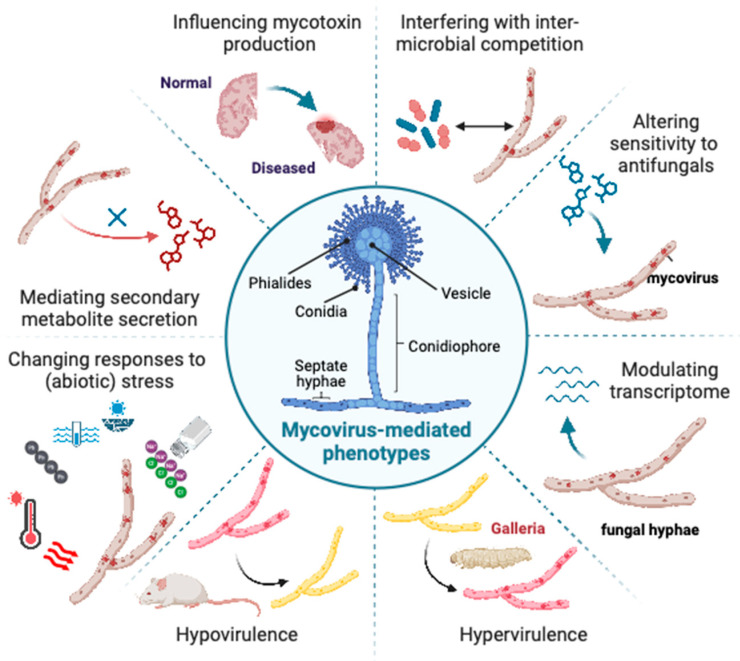
Overview of *Aspergillus* phenotypes altered due to mycovirus infection. Created with Biorender.com.

Mycovirus-mediated phenotypes in *Aspergilli* have been previously reviewed, with alterations in growth, pigmentation, sporulation, toxin production and virulence previously reported [60]. Nonetheless, additional phenotypes, in particular related to stress response, have since been uncovered and, while previous studies focused on phytopathogenic fungi, here we draw attention to mycovirus-mediated phenotypes in human pathogenic *Aspergilli*. A summary of these phenotypes can be found in Table S1 and is illustrated in Figure 2.

### 3.1. Aspergillus fumigatus

The ubiquitous and airborne human pathogen, *A. fumigatus*, poses a significant health risk as the primary agent of potentially fatal invasive aspergillosis infections, which especially affect immunocompromised individuals [55]. The increasing incidence of fungal resistance to antifungal agents, such as azoles, has exacerbated the burden of this pathogen [122]. Mycovirus-mediated virulence modification in *A. fumigatus* has previously been examined in an insect model (*Galleria mellonella*) [105,121], but until recently had not been observed in a vertebrate model.

Takahashi-Nakaguchi et al. [99] reported reduced virulence of an *A. fumigatus* strain infected with AfuCV41362, a new member of the *Chrysoviridae* (see Section 2.2.2, ‘*Chrysoviridae’*), as compared to a virus-free isogenic strain, following infection of immunocompromised mice. The decreased mortality of mice inoculated with the virus-infected strain was accompanied by lower lung fungal burden. To further investigate this, gene expression in isogenic virus-free and virus-infected strains was compared during germination, the stage in which expression of all four AfuCV41362 genes was highest. AfuCV41362 down-regulated expression of numerous stress tolerance genes, including transcripts related to formate and nitric oxide (NO) detoxification and hypoxia adaption. Correspondingly, in vitro analysis indicated that the virus-infected strain had decreased tolerance to formate, hypoxic, NO and oxidative stresses, as well as reduced hydrophobicity. Stress tolerance may be associated with fungal pathogenicity, as it facilitates adaption to environmental stresses and microenvironments experienced during invasive pulmonary infection [123], such as hypoxia [124,125]. For example, several *A. fumigatus* genes typically upregulated during growth under hypoxic conditions, e.g., those encoding proteins involved in NAD+ regeneration, were instead suppressed in the virus-infected strain. Furthermore, mammalian immune cells produce NO to defend against invasive pathogens, which fungi combat *via* upregulation of NO detoxification genes [126]. Several of these NO detoxification genes, such as *fhpA*, which encodes reactive nitrogen species detoxification flavohemoprotein, were downregulated by AfuCV41362. Moreover, suppression of stress tolerance may increase the susceptibility of *A. fumigatus* to the human immune system, producing a less invasive phenotype.

Similarly, AfuPmV1M, a strain of AfuPmV1 from *Polymycoviridae* (see Section 2.2.4, ‘*Polymycoviridae’*), caused reduced mortality during *A. fumigatus* infection in a mouse model [107]. In vitro growth experiments showed that AfuPmV1M decreased mycelial mass and conidia formation, and delayed germination. RNA-seq of an AfuPmV1M-infected strain revealed that genes involved in gliotoxin synthesis were downregulated, while fumagillin synthesis genes were upregulated. These results were corroborated by the respective quantities of gliotoxin and fumagillin accumulated in fungal cultures. Gliotoxin is a mycotoxin with auto-regulatory roles in the fungus, and immunosuppressive and pro-apoptotic activity against mammalian immune cells, rendering it a virulence factor of *A. fumigatus* infection [127,128]. Suppression of gliotoxin biosynthesis, therefore, likely contributes to hypovirulence. Contrastingly, fumagillin, another mycotoxin, is implicated in tissue damage during invasive infection [129], thus the reasons for its enhanced production remain elusive. Furthermore, decreased adherence of virus-infected conidia to pulmonary epithelial cells in vitro, as well as increased sensitivity to phagocytosis by mouse macrophages in vitro was observed. The latter may relate to the observation that virus-infected conidia also exhibited reduced tolerance to oxidative stress; this is because macrophages produce reactive oxygen species (ROS), inducing oxidative stress, as part of their immune response [130]. Taken together, retarded mycelial growth and conidia formation, in combination with impaired adherence to lung epithelium and tolerance of host immune responses, likely underpin this hypovirulence.

In addition to hypovirulence, mycoviruses have previously been implicated in inducing hypervirulence. To our knowledge, the only report of this phenomenon in *Aspergillus* was by Özkan and Coutts [121], who found that AfuPmV1, close relative of AfuPmV1M (see Section 2.2.4, ‘*Polymycoviridae’*) caused mild hypervirulence against the larvae of the greater wax moth, *G. mellonella*. While this phenotype would be undesirable in human or phytopathogenic fungi, there are scenarios where it may be advantageous. For example, some entomopathogenic fungi (fungi that infect insects), such as *Beauveria bassiana* and *Metarhizium anisopliae*, are employed commercially as biological control agents for crop protection from insect pests or insect vector-borne disease control [131,132]; for these strains a hypervirulent phenotype would increase their efficacy as pesticides [48]. Two mycoviruses infecting *B. bassiana*, named Beauveria bassiana polymycovirus 1 (BbPmV1) and Beauveria bassiana non-segmented virus 1 (BbNV1), have been found to induce mild hypervirulence against *G. mellonella* larvae [48]. Mycoviral-enhanced biological control efficacy *via* the induction of hypervirulence could potentially have exciting ramifications for agriculture, ecology, and even public health.

AfuPmV1 has also been found to hinder the resistance of its *A. fumigatus* host in inter-microbial competition with *Pseudomonas* [45]. *A. fumigatus* and *Pseudomonas aeruginosa* co-inhabit, and may compete, in certain ecological niches, including the human microbiome [55]. The respective opportunistic pathogens are frequently found co-residing in the airways of cystic fibrosis or immunocompromised individuals [133,134], meaning changes to their competition may have clinical implications [135]. Both pathogens depend on iron acquisition to survive and depriving their competitor of iron is a key tactic in their competition. To this end, *P. aeruginosa* employs the siderophore pyoverdine [136], as well as the regulator of iron acquisition and virulence Pseudomonas Quinolone Signal (PQS) [137]. Likewise, *A. fumigatus* also uses siderophores for this purpose [138]. Nazik et al. [45] reported that *P. aeruginosa* had a larger inhibitory effect on virus-infected *A. fumigatus* biofilms, compared with virus-free biofilms. The application of iron-scavenging pyoverdine and PQS molecules to the fungal biofilms generated the same effect. Furthermore, application of iron augmented growth of the virus-free fungus compared to virus-infected strains. Together these results indicate that the impaired tolerance to inter-microbial competition of virus-infected *A. fumigatus* may, in part, be attributed to mycovirus-modified iron-metabolism. This hypothesis was further investigated by Patil et al. [40], who measured the extracellular and intracellular siderophore profiles of AfuPmV1-infected *A. fumigatus*, compared with its virus-free counterpart. Their results showed that differences in speed of siderophore mobilization in virus-free as compared to virus-infected strains explained differences in growth, but especially success in the competition for iron. 

Similarly to fungi, bacteria can be infected with their own viruses, known as bacteriophages (phages). Interestingly, a filamentous phage produced by *P. aeruginosa*, known as Pf, modulates biofilm matrix assembly *via* liquid crystalline structures, which affects biofilm assembly and function [139]. Further to this, *P. aeruginosa* phage Pf4 can even inhibit the metabolic activity of *A. fumigatus* biofilms [140]. The findings that Pf4 binds iron and that supplementation of *A. fumigatus* biofilms with iron ameliorates phage-mediated fungal metabolism inhibition indicate that the mechanism of metabolism modulation by Pf4 involves starving *A. fumigatus* of iron [140], similarly to the mycovirus-modified *Aspergillus* iron metabolism previously described [40,45]. Binding of fungal biofilm matrix by bacteriophage, inhibition of biofilm, and a mechanism based on denial of iron, was also shown for *Candida albicans* [141]. The significance of the findings discussed in this paragraph lies in the observation, for the first time, of a virus that can affect physiology without infecting the cell, as well as the potential implications in cystic fibrosis pathology. Bacteriophage–fungal interactions may be a general feature with several pathogens in the fungal kingdom. Whether non-infecting mycoviruses can produce similar effects, external to the fungal cell, needs further investigation.

The potency of common antifungal drugs has also been compared in AfuPmV1-infected *vs*. virus-free *A. fumigatus* [41]. Interestingly, isogenic strains with and without AfuPmV1 displayed similar susceptibilities to the antifungals voriconazole (VCZ) and amphotericin B (AmB), targeting cell membranes, as well as micafungin (MICA) and caspofungin (CASPO), targeting synthesis of cell wall glucans. However, sensitivity to Nikkomycin Z (NikZ), which affects chitin synthesis, was significantly increased in the virus-infected strain. This effect was observed even when the higher basal metabolic rate of the virus-free strain was considered. NikZ, CASPO and MICA all target the cell wall, but whereas NikZ specifically inhibits the chitin synthase enzyme, the latter two drugs suppress beta-1, 3-glucan synthase. Therefore, AfuPmV1 may increase host sensitivity to cell wall stresses, but specifically in relation to chitin synthesis [41]. In turn, this could lead to increased osmotic stress because of compromised cell wall synthesis. Weakened host tolerance to osmotic stress mediated by AfuPmV1, and closely related AfuPmV1M, has been reported [47,107]. Mycoviruses may increase drug susceptibility in a highly specific mechanism-of-action-dependent manner.

Upon further exploration of AfuPmV1-mediated stress tolerance in *Aspergilli*, enhanced sensitivity to the stresses: salt [47], high temperature, hydrogen peroxide (H_2_O_2_), Congo Red (CR) and nutrient deficiency have also been observed [46]. Sass et al. [46] speculated that impaired resistance to high temperature, H_2_O_2_ and CR may all relate to oxidative stress. Firstly, heat shock has been linked to the production of ROS and the induction of oxidative stress in *A. niger* [142]. Secondly, H_2_O_2_ is a ROS and is released by macrophages to induce oxidative stress as part of their immune response to fungi. Lastly, because CR inhibits beta-1, 3-glucan synthase, inhibitors of which were previously shown to have no differential effect on virus-infected *A. fumigatus* [41], its effects are likely related to a different mechanism, unless their targets in the host enzymes are different. CR belongs to a family of compounds, called azo dyes, which have been reported to induce oxidative stress *via* the production of ROS in response to light or reducing agents [46,143,144].

Oxidative stress has been previously implicated in mycovirus-mediated reduced stress tolerance, with reports of ROS tolerance genes down-regulated in AfuPmV1M-infected *A. fumigatus* [107]. Furthermore, CHV1 itself was reported to evoke oxidative stress in its *C. parasitica* host [145]; if this is also true for AfuPmV1, the application of external oxidative stresses (e.g., H_2_O_2_), in combination with suppression of ROS tolerance genes [107], likely underpin the decreased oxidative stress tolerance in virus-infected hosts. That said, the potential viral effects on *Aspergilli* pathways unrelated to oxidative stress should also be explored.

### 3.2. Aspergillus flavus

*A. flavus*, with its unique ability to survive in warmer climates, is among the most prevalent etiological agents of invasive aspergillosis, second only to *A. fumigatus* [146]. In addition, the species contaminates numerous important crops pre- and post-harvest, such as maize, peanuts and cotton, leading to substantial economic losses [147,148]. Following crop infection, *A. flavus* can produce secondary metabolites, including the mutagenic and carcinogenic aflatoxin, which causes aflatoxicosis and chronic illnesses in humans, including liver cancer [149]. Previously, chrysovirus infection of *A. flavus* has been associated with decreased aflatoxin production [150,151]. The soil dwelling species *A. ochraceus* also produces a food-contaminating mycotoxin, namely ochratoxin A (OTA). In contrast to what has been observed in *A. flavus*, infection of *A. ochraceus* with Aspergillus ochraceus virus (AoV), a partitivirus, was found to enhance OTA production [152]. The mechanisms behind this phenotype were unclear, although gene expression analysis suggested that OTA synthesis in the virus-infected strain could employ a biosynthesis pathway that differs from the virus-free strain. Moreover, while overproduction of OTA may bestow the fungus with an evolutionary advantage, the phenotype has negative implications for food production [152].

An association between mycotoxins and human cancer has been well established [153], suggesting mycoviruses that modulate mycotoxin production may have a role in cancer epidemiology [154]. Remarkably, a tentative association between cancer and *Aspergillus* mycoviruses, unrelated to mycotoxin production, has recently been observed [155,156]. The plasma of patients in full remission from acute lymphoblastic leukaemia (ALL) were reported to harbour antibodies against a particular mycovirus-infected *A. flavus* strain, which did not produce aflatoxin [155]. Furthermore, in vitro exposure of mononuclear cells from the patients to the virus-infected strain led to the regeneration of ‘cell surface phenotypes and genetic markers’ indicative of ALL, which was not observed in controls [155]. The findings of this study allude to a potential mycotoxin-independent carcinogenic effect of mycovirus-containing *Aspergilli*, but this awaits independent confirmation in other laboratories.

AfPV1, a novel member of the *Partitiviridae* (see Section 2.2.1, ‘*Partitiviridae’*) was recently reported to induce aberrant colony morphology, retarded growth on certain media, and decreased sporulation in *A. flavus* [82]. These phenotypes were also observed in AfPV1-infected *A. flavus* in a later study by Jiang et al. [88], in addition to larger vacuoles, which may underpin the abnormal colony morphology. AfPV1 infection was also found to increase fungal susceptibility to osmotic and oxidative stress, similarly to AfuPMV1, as well as UV stress. Furthermore, the virus induced hypovirulence in insect (*G. mellonella*), mouse, and plant (*Zea mays*) infection models, as well as reducing conidial adhesion to lung epithelium and enhancing sensitivity to mouse macrophages. As hypothesised in *A. fumigatus*, this hypovirulence could be a result of suppressed stress tolerance. Interestingly, the satellite dsRNA associated with helper AfPV1 virus observed by Jiang et al. [88] (see Section 2.2.1, ‘*Partitiviridae’*), was found to attenuate these phenotypes. Attenuation or exacerbation of virus-mediated phenotypes by satellite viruses has been well documented in plants [157], such as the tobacco necrosis satellite virus, which decreases the pathogenicity of tobacco necrosis virus [84]. However, prior to the Jiang et al. [88] paper, this had not been observed in fungi. The satellite dsRNA putatively reduces the virulence of its helper AfPV1 virus *via* a mechanism that decreases the accumulation of the helper virus in the *A. flavus* host [88].

### 3.3. Factors Affecting Manifestation of Mycovirus-Mediated Phenotypes

Mycovirus-host interactions are complex, and several factors have been reported to influence the manifestation of virus-mediated phenotypes in their fungal host. For example, the novel AthCV1, a chrysovirus (see Section 2.2.2, ‘*Chrysoviridae’*), was found to induce a switch from asexual (conidiospore) to sexual spore (ascospore) production in *A. thermomutatus*–however, the effect of the virus on sporulation was temperature dependent [32]. At 20 °C, conidiation in the AtCV1-infected strain was significantly lower than the virus-free strain, but at 37 °C it was significantly greater [32]. Similarly, AfuPMV1 (see Section 2.2.4, ‘*Polymycoviridae*’) hindered the growth rate of hyphae derived from infected *A. fumigatus* conidia as compared to virus-free strains at 37 °C and at 46 °C, however no virus-mediated growth disadvantaged was reported at 22 °C or when cultures were previously exposed to high and low temperatures [46]. Additionally, some AfuPMV1-mediated phenotypes are also dependent on fungal life stage, with weakened tolerance to high temperature and H_2_O_2_ stress reported in virus-infected *A. fumigatus* cultures started from conidia but not from hyphae [46]. This suggests that fungal stress resistance is compromised by AfuPMV1 predominantly during germination and hyphal development.

It may even be possible that phenotypes manifest differently dependent on the organism infected by the *Aspergillus*. For example, AfuPmV1M-mediated hypovirulence was recently observed in infected adult mice [107], while a similar virus, AfuPmV1, induced hypervirulence in *G. mellonella* larvae [121]. It is possible that the opposite effects on virulence observed in these studies relates to the virus’s evolutionary “strategy”. Mycoviruses rely solely on their fungal host for transmission; therefore, because adult mice are mobile, prolonging mouse survival time upon infection by fungi may increase the likelihood that the virus spreads geographically [158]. Conversely, prolonged host survival upon fungal infection would not be beneficial to the virus in larvae, because the larvae are immobile. Future studies may wish to compare the effects of the same mycovirus and fungal host in larvae vs. adults of the same insect species to investigate this hypothesis further. Nonetheless, AfuPmV1 and AfuPmV1M differ in the possession of the fifth dsRNA segment, which may instead harbour a gene function responsible for this difference in virus-mediated virulence modulation.

A recent study by Kuroki et al. [83] has demonstrated the highly specific virus- and host-strain dependent manner in which mycovirus-mediated phenotypes manifest. In this study, three partitiviruses of the same species, genetically most similar to AfPV1, were isolated from separate strains of *A. flavus* and the number of differentially expressed genes was recorded for each virus-infected strain compared with their virus-free isogenic counterpart. Interestingly, the percentage of differentially expressed genes shared across the three partitivirus-infected strains was minimal, providing evidence against a generic partitivirus transcriptomic response [83]. Moreover, even if the virus and host species are the same, a different transcriptional response may be induced, suggesting that fungal response to viral infection is specific to the mycovirus-host combination. To investigate further, Kuroki et al. [83] designed a virus-swapping experiment, in which two partitiviruses, with genomes varying at only three nucleotides, were swapped between their original genetically similar *A. flavus* hosts. While no altered phenotypes were observed, gene expression was notably dissimilar in the virus-swapped isolates compared to the natural host isolates, demonstrating that sequence variation at just three nucleotides can evoke a differential host transcriptional response. Similarly, infection of different *A. flavus* hosts with genetically identical partitiviruses also induced differential gene expression. Taken together, a unique relationship, and perhaps compatibility, exists between mycoviruses and their natural host.

Another factor in the manifestation of mycoviral phenotypes is co-infection of a fungal host with multiple viruses. Kuroki et al. [83] isolated an *A. flavus* strain infected simultaneously with a partitivirus and a polymycovirus. The authors produced isogenic strains of this fungus, singly infected with each of the viruses. Interestingly, they observed phenotypic alterations in the co-infected strain, including enhanced UV tolerance, decreased conidiation, altered aerial hyphae morphology and enhanced secondary metabolite production, which were not reported in the singly infected strains. Notably, the co-infected strain displayed the greatest proportion of differentially expressed genes as compared with singly infected strains.

## 4. Molecular Mechanisms Underpinning Mycovirus-Mediated Phenotypes

### 4.1. RNA Silencing: Antiviral Defence in Fungi

As described in Section 3, ‘Mycovirus-mediated phenotypes’, mycoviruses can modulate the transcriptome of their fungal host, which in some instances leads to an altered phenotype. However, further insight into the viral and host interactions that cause these modifications remain elusive. Most prominently, the role of RNA silencing in evoking mycoviral phenotypes has been explored. RNA silencing is a conserved defence mechanism against mobile genetic elements, such as viruses and transposons, and has been well reported in animals and plants [159,160,161]. The mechanism involves the endoribonuclease Dicer, which recognises and cleaves viral dsRNA genomes or replicative intermediates, generating virus-derived small interfering RNA (vsiRNA) [162]. Subsequently, vsiRNAs are incorporated with Argonaute proteins to form the RNA-induced silencing complex (RISC), which targets and degrades complementary mRNA [73]. The role of RNA silencing in fungal antiviral defence was first reported in *C. parasitica*, whereby CHV1-infected RNA silencing machinery mutants displayed severe debilitation symptoms, as compared to virus-free mutants [163,164]. Essentially, there are two avenues through which mycoviruses may potentially use RNA silencing to induce host transcriptomic and phenotypic alterations. Firstly, if RNA silencing plays a role in modulating host gene expression, then mycovirus-mediated activation or suppression of the mechanism will disrupt functionality of these host genes [73]. Secondly, if enough homology exists between vsiRNA and host RNA, RISC may target host, as well as viral, transcripts [73,165].

Orthologous components of the RNA silencing pathway have been observed across the kingdom Fungi [166]. In the model filamentous fungus *C. parasitica*, the dicer-like 2 (*dcl2*) and Argonaute-like 2 (*agl2*) genes are indispensable for antiviral RNA silencing and become transcriptionally upregulated in response to mycoviral infection [163,164,167,168]. The process behind mycoviral-activation of these genes, however, is less well understood. Andika et al. [169] discovered that the conserved transcriptional coactivator Spt–Ada–Gcn5 acetyltransferase (SAGA) complex is a regulator of *dcl2* and *agl2* upregulation in *C. parasitica.* Remarkably, DLC2, in addition to its enzymatic activity in dsRNA cleavage for RNA silencing, also facilitates the SAGA-induced upregulation of a diverse set of fungal host genes in response to mycovirus infection [170]. This includes the upregulation of *dcl2* and *agl2*, inducing a positive feedback loop, as well as genes involved in amelioration of symptoms induced by the virus [170]. Moreover, DCL2 contributes to fungal antiviral defence at both the transcriptional and post-transcriptional level and, while it remains unknown if the same mechanism operates in *Aspergillus*, this finding helps illustrate how mycoviral activation of RNA silencing plays a role in modifying expression of a range of genes in the fungal host.

#### 4.1.1. Mycoviruses Are Triggers, Targets and Suppressors of RNA Silencing

In *Aspergillus*, mycoviruses can be both triggers and targets, as well as suppressors, of RNA silencing. This was initially demonstrated by Hammond et al. [73], who reported that (i) AsV1816 suppressed RNA silencing in *A. nidulans*, *via* a mechanism that decreased the number of vsiRNAs, while (ii) Aspergillus virus 341 was targeted for cleavage by RNA silencing, evidenced *via* the presence of vsiRNAs. Similarly to the latter observation, sRNA sequencing by Özkan et al. [162] showed that mycoviruses AfuPmV1, AfuPV1 and AfuCV (see Section 2.2, ‘Expansion of the dsRNA virome’) are also targeted by *A. fumigatus* RNA silencing machinery and are processed into vsiRNAs. Interestingly, production of vsiRNAs in this investigation occurred concurrently with silencing of *A. fumigatus* genes that had high sequence similarity to the viral transcripts, illustrating how the RNA silencing mechanism may cause off-target transcriptome modification of the fungal host [162].

#### 4.1.2. RNA Silencing Suppression Mechanisms

A recent study by Jiang et al. [171] has shown that AfPV1 may function as an RNA silencing suppressor in its *A. flavus* host. Using RT-qPCR, the authors initially demonstrated that expression of RNA silencing components was upregulated in *A. flavus* in response to AfPV1 infection. However, a subsequent reverse genetics experiment showed that virus-infected mutant strains, in which components of RNA silencing had been deleted, displayed decreased accumulation of AfPV1 RNAs compared to virus-free mutants. This observation is unexpected considering the role of RNA silencing components in antiviral defence, thereby suggesting the virus encodes an RNA silencing suppressor. At present, few studies have elucidated the mechanisms by which mycoviruses are able to suppress RNA silencing, but these could involve protection of dsRNA, inhibition of Dicer and Argonaute proteins, or high turnover of vsiRNAs [73]. Identified mycoviral RNA silencing suppressors include (i) p29 encoded by CHV1, which inhibits transcription of integral RNA silencing genes in *C. parasitica* [172], and (ii) p20 encoded by Fusarium graminearum hypovirus 1 (FgHV1), which binds single-stranded small interfering RNA (siRNA) in *F. graminearum* [173], among other mechanisms [174,175,176].

### 4.2. Function of Individual Viral Proteins

A central facet in the study of the mechanisms behind mycovirus-mediated phenotypes is examining the functions of individual ORFs, and their encoded proteins, within a mycovirus. As such, to understand how AfuCV41362 caused hypovirulence in its *A. fumigatus* host (see Section 3, ‘Mycovirus-mediated phenotypes’), Takahashi-Nakaguchi et al. [99] ectopically expressed each of the virus’s four ORFs individually in a natively virus-free strain. Ectopic expression of ORF1 enhanced germination but decreased mycelial mass in its *A. flavus* host. ORF1 is identical to the virus originally designated as AsV1816, which was found to suppress RNA silencing in *A. nidulans* [73], suggesting that a similar function may be involved in producing the AfuCV41362-mediated phenotypes. Despite this, expression levels of RNA silencing components Dicer and Argonaute were unchanged in AfuCV41362-infected and virus-free strains, indicating a different mechanism is involved. Furthermore, ectopic expression of ORF3 caused decreased conidial numbers, as well as reduced stress tolerance. Likewise, ORF2 and ORF4 also reduced tolerance to stress. Notably, ectopic expression of each ORF individually was unable to significantly restore hypovirulence in the mouse model. Taken together, the authors suggest that AfuCV41362-mediated hypovirulence likely occurs *via* a mechanism independent of RNA silencing, involving cooperation between each ORF.

A similar experiment was carried out by Takahashi- Nakaguchi et al. [107] to investigate the function of individual AfuPmV-1M ORFs producing hypovirulence in their *A. fumigatus* host (see Section 3, ‘Mycovirus-mediated phenotypes’). Their results also suggested a synergy between segments, reporting that ORF2 and ORF5 reduced fungal virulence in a mouse model, while ORF3 reduced fungal stress tolerance. The findings that ORF5 induced decreased virulence may also explain why AfuPmV1, a close relative of AfuPmV1M that lacks ORF5, was previously reported to have no effect [105] or a hypervirulent effect [121] on fungal virulence in an insect larvae model. To further uncover the mechanisms of mycovirus-mediated phenotypes, future studies should examine the host protein-viral protein interactions of these individual ORFs to elucidate the biochemical pathways involved in producing these phenotypes.

Recent efforts by our group, not yet published in full, portend important insights into the mechanisms, already described in this review, of AfuPmV1 effects, and full peer-reviewed publications are expected in short order. A profound role of the virus in gliotoxin release has been shown [177,178]. Furthermore, the alteration of specific *Aspergillus* virulence factors has been demonstrated with proteomic analysis [179]. The specific *Aspergillus* genes that are affected by the virus in its modulation of fungal iron metabolism have also been defined [180].

## 5. Mycovirus-Based Applications: A Role in Therapeutics?

The growing global health burden of invasive fungal infections (IFI), driven predominantly by increasing numbers of immunocompromised individuals, toxicity of existing antifungal drugs and emerging antifungal resistance, has placed significant pressure on the search for alternative IFI treatments [181,182,183]. Multi-drug resistant (MDR) bacteria have caused a similar pressure for bacterial infections and recently there has been a resurgent interest in the use of bacteriophages to treat bacterial infections. Phage therapy employs lytic phages, which replicate in their host cell before lysing it. In addition to its application in cases of, or to avert, MDR, phage therapy is also highly bacterial strain-specific and thus does not target the total body microbiota [184]. Furthermore, phage therapy can be used to treat biofilms, such as in the lungs of cystic fibrosis patients [185]. Although pre-clinical studies have successfully demonstrated phage activity against human pathogenic bacteria, such as *P. aeruginosa*, *E. coli*, *Klebsiella pneumoniae* and *Achromobacter xylosoxidans*, in murine and human models [185,186,187,188,189], robust clinical trials, safety testing, scalable manufacturing processes and regulations for phage therapy implementation have not yet been put in place [190]. Nonetheless, as the virome in human pathogenic *Aspergilli* expands [28], it has become increasingly viable that, in an approach analogous to phage therapy, mycoviruses could be employed to treat IFIs.

As delineated in a thorough review by van de Sande et al. [191], there are several requirements for the application of a mycovirus as a therapeutic. Most prominently, the mycovirus would require an extracellular life cycle phase, to facilitate its delivery (*via* inhalation or injection) to the site of fungal infection. At present, the majority of known mycoviruses can only be transmitted intracellularly as their genome sizes are greater than that of the microscopic fungal cell wall pores [192]. Delivery of the virus in the absence of an extracellular phase would require inoculating the patient with virus-infected fungi, to facilitate virus transmission *via* hyphal anastomosis. This approach is possibly dangerous as increasing a patient’s fungal lung burden carries further risks. Excitingly, the first fungal ssDNA virus, namely Sclerotinia sclerotiorum hypovirulence-associated DNA virus 1 (SsHADV1) from the phytopathogenic fungus *Sclerotinia sclerotiorum*, was found to have extracellular routes of transmission [193,194]. Moreover, screening of *Aspergilli* for ssDNA may be a logical starting point in the search for mycoviral therapeutic candidates that transmit extracellularly. Furthermore, the ability of the SsHADV1 CP to transmit genetic material extracellularly could be exploited to produce a genetically engineered vector, carrying heterologous nucleic acids (i.e., therapeutic *Aspergilli* mycoviruses), and delivering these to target fungi (i.e., *Aspergilli* in the lungs). Finally, liposomal carriers, possibly even in liposomal amphotericin B (for dual therapy), could be employed to encase and deliver mycoviruses to fungal cells [191,195].

Upon successful delivery to the lungs, a therapeutic mycovirus must sufficiently debilitate the fungus so that infection in the patient ceases or is at least reduced; induction of hypovirulence or a killer phenotype are two mechanisms through which that could be achieved [191]. Mycoviruses that induce hypovirulence in *A. fumigatus* have been observed in a murine model [99,107], presenting potential candidates for therapeutics once their efficacy in human models is examined. Despite being reported in many fungal species since the first observation in *S. cerevisiae*, no mycoviruses inducing killer phenotypes have been isolated yet in *Aspergillus* [196]. Nonetheless, killer toxins from other fungal species have displayed growth inhibition activity against a range of *Aspergillus* species, such as toxins K3 and PakT from *Wickerhamomyces anomalus*, and can be isolated from their host and applied as a therapeutic without requiring the virion [191,197]. For example, lesions produced by superficial *Malassezia furfur* infection in guinea pigs were reduced by topical application of isolated PakT [198]. Moreover, research efforts to delineate the mycoviral mechanisms causing hypovirulence or killer systems, may enable the production of genetically engineered viruses that induce these desired debilitation phenotypes.

The final requirement for a therapeutic mycovirus is low antigenicity; the mycoviruses or killer toxin must not trigger the innate or adaptive immune system of the human host. In mammals, viral dsRNAs are recognised by proteins called pattern recognition receptors (PRRs), such as mammalian Toll-like receptor 3 (TLR3) and retinoic acid inducible gene-I (RIG-I)-like receptors (RLR). Activation of PRRs by dsRNA triggers a signalling cascade, culminating in innate immune responses, such as activation of transcription factors, the expression of pro-inflammatory cytokines and interferons, and, ultimately, cell apoptosis. The discovery of *Aspergillus*-derived virus particles capable of inducing the production of interferons in mammalian cells upon fungal infection dates back 50 years [59]. There is an extensive body of basic research on interferon as a therapeutic against several fungal infections in vitro and in animal models [199]. Further research is required to elucidate the extent to which mycoviruses trigger the mammalian innate immune response and the resultant impact (positive or negative) of this on the mammalian host. For example, a totivirus within the protozoan parasite *Leishmania guyanensis* is recognised by TLR3 following infection with the parasite, launching a hyper-inflammatory response, and amplifying the host’s susceptibility to the infection [200,201]. As for an example in fungi, a totivirus in *Malassezia restricta* was found to drive the production of cytokines in a TLR3-induced inflammatory response in mouse bone marrow-derived dendritic cells during fungal infection [202]. Furthermore, killer toxins are known to have high antigenicity and can be toxic to the host of the fungal infection, for instance PakT (from *W. anomalus*), was found to cause debilitating injury to the small intestine in a murine model [203]. To circumvent this, the use of anti-idiotypic antibodies that imitate killer toxins have been explored. Notably, killer toxin anti-idiotypic antibodies against *A. fumigatus* were found to attenuate infection in a murine model [204]. Moreover, the potential human immune response to specific mycovirus strains or their killer toxins must be considered prior to their application as therapeutics.

## 6. Conclusions and Future Perspectives

NGS and RNA-seq have facilitated the identification of mycoviruses from a broader host range within *Aspergillus*, as well as illustrating the previously underestimated diversity of the fungal virome, with peculiar and interesting genome structures being increasingly isolated. As understanding of the *Aspergillus* virome expands, so has our knowledge of the extent to which mycoviruses influence phenotypes of their fungal host, such as affecting virulence and reducing stress tolerance. Research has begun to uncover, in part, the mechanisms behind mycovirus-mediated phenotypes, such as the nuanced manner in which viruses may be triggers, targets and even suppressors of RNA silencing and the effect this has on global fungal gene expression. Nonetheless, to unlock the full potential of *Aspergilli* mycoviruses, these mechanisms require full delineation.

A redirection of mycovirus research from more traditional methods to reverse genetics systems based on the use of infectious viral complementary DNA (cDNA) clones and in vitro transfection has the potential to reshape our understanding of the causal relationship between mycoviral gene sequences and fungal phenotypes [205]. In turn, identification of viral genomic determinants for specific host phenotypes may facilitate the construction of infectious viral clones, in which user-defined mutations leading to desired fungal phenotypes, can be introduced. Similarly, infectious clones may be used as vectors for virus-induced gene silencing (VIGS). This approach has already proved successful using the Fusarium graminearum gemytripvirus 1 (FgGMTV1) to assemble a VIGS vector that silenced virulence genes in the agriculturally destructive cereal pathogen *Fusarium graminearum*, resulting in a protective effect on wheat [206]. Furthermore, circumvention of the VI-imposed virus transmission barrier by in vitro transfection may expand fungal host ranges, allowing phenotypes to be introduced in specific hosts for desired outcomes. While such synthetic biology approaches have not yet been established for *Aspergillus*, the development of reverse genetics systems for mycoviruses of several other ascomycetes [35,205,206,207,208,209,210] is promising for future research.

## Data Availability

Data underlying this review is provided in the extensive Reference sect. appended.

## Supplementary Materials

The following supporting information can be downloaded at: https://www.mdpi.com/article/10.3390/jof10080585/s1. Table S1: Mycoviruses isolated from Aspergillus since 2017 and their properties.
