# Supplementary material for: The Expanding Mycovirome of Aspergilli
Source: J Fungi (Basel). 2024 Aug 17;10(8):585. doi: 10.3390/jof10080585 (PMC11355518; doi:10.3390/jof10080585)
Supplement: Supplementary file 1 [file jof-10-00585-s001.zip › jof-3102889-supplementary.pdf]

Table S1: Mycoviruses isolated from Aspergillus since 2017 and their properties. Mycovirus isolated prior to 2017 have not been included here, but can be found in a previous review [60]. The genome type and taxonomic ranks family and genus or each virus are given, along with the *Aspergillus* strain it was isolated from. For each viral RNA segment, size is provided in base pairs (bp), as well as the size of the protein encoded in amino acids (aa) and kilo Daltons and its putative function. Additionally, reported effects of the mycovirus on fungal host phenotype are summarised and the method used to detect the virus is displayed.

Genome type	Family	Genus	Mycovirus	Host	RNA segment	RNA segment size (bp)	Accession number (nucleotide/ protein)	Protein encoded (aa/kDa)	Detection method	Effect on host phenotype	Reference
**(+) ssRNA**	***Narnaviridae***	*Narnavirus*	Aspergillus fumigatus narnavirus 1 (AfuNV1)	*A. fumigatus* V145-43	RNA1	2007	MH192989/ AXE72933	RNA-dependent RNA polymerase (618/107)	RNA-seq	Unknown	[62]
			Aspergillus fumigatus narnavirus 2 (AfuNV2)	*A. fumigatus* V145-80, V145-33	RNA1	1994	MH192990/ AXE72934	RNA-dependent RNA polymerase (626/70.7)	RNA-seq	Unknown	[62]
				A. fumigatus IFM 63147	RNA1	2008	LC553693/ BCH36633	RNA-dependent RNA polymerase (626/70.7)	RNA-seq	Unknown	[63]
					RNA2	2118	LC553694/ BCH36634	Hypothetical protein (649/76)			
					RNA3	1301	LC553695/ BCH36635	Hypothetical protein (159/17.8)			
		Unclassified *Narnaviridae*	Aspergillus lentulus narnavirus 1 (AleNV1)	*A. lentulus* IFM 63547	RNA1	3074	LC553702/ BCH36643	RNA-dependent RNA polymerase (983/110.5)	RNA-seq	Unknown	[63]
					RNA2	1817	LC553703/ BCH36644	Hypothetical protein (236/25)			
			Aspergillus tennesseensis narnavirus 1 (AtenNV1)	*A. tennesseensis* #1383_1_1	RNA1	1861	LC648937/ BDB16250	RNA-dependent RNA polymerase (556/62.5)	RNA-seq	Unknown	[64]
					RNA2	1682	LC648938/ BDB16251	RNA-dependent RNA polymerase (478/53.8)			
					RNA3	1311	LC648939/N A	N/A			
					RNA4	740	LC648940/ NA	N/A			
					RNA5	684	LC648941/ NA	N/A			

			Aspergillus flavus narnavirus 1 (AfNV1)	*A. flavus* Lom7, MN1,	RNA1	2102	MZ600054/ UAW09566	RNA-dependent RNA polymerase (657/75.9)	RNA-seq	Unknown	[66]
					RNA2	2092	MZ600055/ UAW09567	Replicase (657/ 74.4)			
					RNA3	1294	MZ600056/ UAW09580	Hypothetical protein (195/22.2)			
			Aspergillus flavus narnavirus 2 (AfNV2)	*A. flavus* Pie 6, Ven7	RNA1	3486	MZ600057/ UAW09568	RNA-dependent RNA polymerase (1092/119.8)	RNA-seq	Unknown	[66]
			Aspergillus creber narnavirus 1 (AcreNV1)	*A. creber* SC-f7 F59	RNA1	3409	LC648933/ BDB16246	RNA-dependent RNA polymerase (1076/124.2)	RNA-seq	Unknown	[64]
					RNA2	1898	LC648934/ BDB16247	Hypothetical protein (287/32.2)			
					RNA3	1653	LC648935/ BDB16249	Hypothetical protein (244/26.4)			
					RNA4	1073	LC648936/ NA	N/A			
	Mitoviridae	*Mitovirus*	Aspergillus fumigatus mitovirus 1 (AfuMV1)	*A. fumigatus* V145-33	RNA1	2500	MH192988/ BCH36632	RNA-dependent RNA polymerase (587/68.7)	RNA-seq	Unknown	[62]
	Botourmiaviridae	Unclassified *Botourmiaviridae*	Aspergillus fumigatus botourmiavirus 1 (AfuBOV1)	*A. fumigatus* IFM 63147, IFM 64916, IFM 64779	RNA1	2399	LC671624/ BDF97672	RNA-dependent RNA polymerase (660/75)	RNA-seq	Unknown	[28]
			Aspergillus pseudoviridinutans botourmiavirus 1 (ApvBOV1)	*A. pseudoviridinutans* IFM 59502, IFM 59503, IFM 61377, IFM 61378	RNA1	2900	LC553709/ BCH36654	RNA-dependent RNA polymerase (626/71.3)	RNA-seq	Unknown	[63]
		Magoulivirus	Aspergillus flavus magoulivirus 1 (AfMoV1)	*A. flavus* CR1	RNA1	2863	MZ600058/ UAW09569	RNA-dependent RNA polymerase (680/77.5)	RNA-seq	Unknown	[66]
		Scleroulivirus	Aspergillus flavus scleroulivirus 1 (AfSoV1)	*A. flavus* CR5	RNA1	3009	MZ600059/ UAW09570	RNA-dependent RNA polymerase (716/81.3)	RNA-seq	Unknown	[66]

		Ourmiavirus	Aspergillus creber ourmiavirus 1 (AcreOV1)	*A. creber* NT10-19-SO2-15	RNA1	2762	LC648942/ BDB16252	RNA-dependent RNA polymerase (747/84.3)	RNA-seq	Unknown	[64]
					RNA2	892	LC648943/ NA	N/A			
**dsRNA**	***Partitiviridae***	Unclassified *Partitiviridae*	Aspergillus fumigatus partitivirus 1 (AfuPV1)	*A. fumigatus* 12-43	RNA1	1779	FN376847/ CAY25801	RNA-dependent RNA polymerase (542/63)	dsRNA extraction	Unknown	[80][81]
					RNA2	1623	FN398100/ CAZ61323	Coat protein (442/48)			
					RNA3	1112	LR746165/ CAA7351346	Unknown (258/29.3)			
			Aspergillus fumigatus partitivirus 2 (AfuPV2)	*A. fumigatus* V145-13, V145-40	RNA1	1822	MH192991/ AXE72935	RNA-dependent RNA polymerase (573/65.6)	RNA-seq	Unknown	[62]
					RNA2	1638	MH192992/ AXE72936	Coat protein (483/54.8)			
			Aspergillus lentulus partitivirus 1 (AlePV1)	*A. lentulus* IFM 62627	RNA1	1820	LC553700/ BCH36641	RNA-dependent RNA polymerase (582/66.5)	RNA-seq	Unknown	[63]
					RNA2	1646	LC553701/ BCH36642	Coat protein (483/54.8)			
			Aspergillus flavus partitivirus 1 (AfPV1)	*A. flavus* LD-3-8, ZD1.22-10-9	RNA1	1764	MK344768/ QDE53634	RNA-dependent RNA polymerase (548/63.4)	dsRNA extraction	Virulence ↓<br>Growth ↓<br>Vacuole size ↑<br>sporulation ↓<br>Osmotic, oxidative and UV stress tolerance ↓	[82][88]
					RNA2	1391	MK344769/ QDE53635	Hypothetical protein (369/42.2)			
					RNA3	1187	MK344770	Hypothetical protein (281/32.2)			
					Satellite RNA	734	OM650825/ NA	N/A			
			Aspergillus nidulans partitivirus 1 (AnPV1)	*A. nidulans* strain HJ5-47	RNA1	1837	MW002435/ QQZ02313	RNA-dependent RNA polymerase (573/65.8)	dsRNA extraction	Unknown	[92]
					RNA2	1583	MW002436/ QQZ02314	Capsid protein (489/54.7)			

			Aspergillus protuberus partitivirus 1	*A. protuberus* LIMA-18-5_MYP	RNA1	1770	LC648931/ BDB16244	RNA-dependent RNA polymerase (539/62.2)	RNA-seq	Unknown	[64]
					RNA2	1570	LC648932/ BDB16245	Hypothetical protein (439/47.2)			
			Aspergillus creber partitivirus 1 (AcrePV1)	*A. creber* SC-f7 F59	RNA1	1821	LC648929/ BDB16242	RNA-dependent RNA polymerase (571/65)	RNA-seq	Unknown	[64]
					RNA2	1542	LC648930/ BDB16243	Hypothetical protein (470/52.9)			
		Gammapartitivirus	Aspergillus niger partitivirus 1 (AnPV1)	*A. niger* clinical isolate	RNA1	1741	LC671611/ BDF97658	RNA-dependent RNA polymerase (542/62.5)	RNA-seq	Unknown	[28]
					RNA2	1368	LC671612/ BDF97659	Capsid protein (371/41.4)			
	Chrysoviridae	*Unclassified chrysovirus*	Aspergillus cibarius chrysovirus 1 (AcCV1)	*A. cibarius* NW-FVA 2590	RNA1	3683	ON033147/ WBW48474	RNA-dependent RNA polymerase (1118/126.7)	dsRNA extraction	Unknown	[94]
					RNA2	3093	ON033148/ WBW48475	Capsid protein (905/100.8)			
					RNA3	2902	ON033149/ BW48476	Hypothetical protein (842/94)			
		Betachrysovirus	Aspergillus thermomutatus chrysovirus 1 (AthCV1)	*A. thermomutatus* clinical isolate	RNA1	3589	MF045841/ AWC67507	RNA-dependent RNA polymerase (1114/123.7)	dsRNA extraction	Sporulation ↓	[32]
					RNA2	2772	MF045842/ AWC67508	Capsid protein (825/100.8)			
					RNA3	2676	MF045843/ AWC67509	Hypothetical protein (768/88)			
					RNA4	2514	MF045844/ AWC67510	Hypothetical protein (711/75.8)			
		Alphachrysovirus	Aspergillus terreus chrysovirus 1 (AtCV1)	*A. terreus* HJ3-26	RNA1	3612	OQ830494/ WKU83718	RNA-dependent RNA polymerase (1124/130)	dsRNA extraction	Unknown	[95]
					RNA2	3132	OQ830495/ WKU83719	Capsid protein (971/107.6)			

					RNA3	3153	OQ830496/ WKU83721	Hypothetical protein (841/94.1)			
					RNA4	3144	OQ830497/ WKU83722	Hypothetical protein (924/103.5)			
			Aspergillus fumigatus chrysovirus 41362 (AfuCV41362)	*A. fumigatus* strain IFM 41362	RNA1	3564	LC350094/ BBC45614	RNA-dependent RNA polymerase (1134/127.3)	dsRNA extraction	Virulence ↓<br><br>Formate, hypoxia, NO, hydrophobicity and oxidative stress tolerance ↓	[99]
					RNA2	2863	LC350095/ BBC45615	Unknown (783/85.7)			
					RNA3	2728	LC350096/ BBC45616	Coat protein (762/82.5)			
					RNA4	2611	LC350097/ BBC45617	Unknown (704/76.2)			
	Totiviridae	*Victorivirus*	Aspergillus niger victorivirus 1 (AnV1)	*A. niger* strain baiyun3.23-4	RNA1	5317	OM293970/ ORF1:UPO09357 ORF2:UPO09358	ORF1: Coat protein (779/80.80) ORF2: RNA-dependent RNA polymerase (827/91.1)	dsRNA extraction	Unknown	[102]
		Unclassified *Totiviridae*	Aspergillus lentulus totivirus 1 (AleTV1)	*A. lentulus* IFM 65052	RNA1	5171	LC553706/ ORF1: BCH36650 ORF2: BCH36651	ORF1: Coat protein (776/80) ORF2: RNA-dependent RNA polymerase (826/89.8)	RNA-seq	Unknown	[63]
	Polymycoviridae	*Polymycovirus*	Aspergillus fumigatus polymycovirus 1M (AfuPmV1M) *	*A. fumigatus* Af293	RNA1	2403	LC517041/ BBU42080	RNA-dependent RNA polymerase (763/84.1)	dsRNA extraction	Virulence ↓<br><br>Mycelial mass ↓<br><br>Conidia formation ↓<br><br>Gliotoxin synthesis ↓	[107]
					RNA2	2230	LC517042/ BBU42081	Hypothetical protein (696/75.7)			
					RNA3	1971	LC517043/ BBU42082	Methyl transferase (622/66.7)			

					RNA4	1156	LC517044/ BBU42083	Hypothetical protein (297/30.7)		Fumagillin synthesis ↑<br><br>Oxidative stress tolerance ↓	
					RNA5	1140	LC517045/ BBU42084	PAS-rich protein (279/29)			
			Aspergillus spelaeus polymycovirus 1 (AsPmV1)	*A. spelaeus* MUT1993	RNA1	2384	MG887754/ AYP71805	RNA-dependent RNA polymerase (760/83.4)	RNA-seq	Unknown	[106]
					RNA2	2219	MG887757 AYP71808	Hypothetical protein (695/65.6)			
					RNA3	1968	MG887756/ AYP71807	Methyl transferase (448/48.9)			
					RNA4	1194	MG887755 AYP71806	PAS-rich protein (172/18.2)			
			Aspergillus flavus polymycovirus 1 (AfPMV1)	*A. flavus* CR1, CR9	RNA1	2394	MZ600062/ UAW09573	RNA-dependent RNA polymerase (760/83.4)	RNA-seq	Unknown	[66]
					RNA2	2257	MZ600065/ UAW09576	Hypothetical protein (776/87.7)			
					RNA3	1931	MZ600064/ UAW09575	Methyl transferase (612/67.5)			
					RNA4	1222	MZ600063/ UAW09574	PAS-rich protein (312/33.6)			

* Aspergillus fumigatus polymycovirus 1 (AfuPMV1), close relative of Aspergillus fumigatus polymycovirus 1M lacking dsRNA5, is not shown in this table as it has been included in a previous review (Kotta-Loizou and Coutts, 2017). AfuPMV1-mediated phenotypes differ from that of AfuPMV1M, see section 'Mycovirus-mediated phenotypes'.
